# Comparison of endoscopic naso-gallbladder drainage and percutaneous transhepatic gallbladder drainage in acute suppurative cholecystitis: Study Protocol Clinical Trial (SPIRIT Compliant)

**DOI:** 10.1097/MD.0000000000019116

**Published:** 2020-02-21

**Authors:** Peilei Mu, Ping Yue, Tianya Li, Bing Bai, Yanyan Lin, Jinduo Zhang, Haiping Wang, Ying Liu, Jia Yao, Wenbo Meng, Xun Li

**Affiliations:** a The First Clinical Medical School of Lanzhou University; b Department of Special Minimally Invasive Surgery, The First Hospital of Lanzhou University; c Gansu Province Key Laboratory of Biological Therapy and Regenerative Medicine Transformation; d Foreign Languages Department of Lanzhou University; e Clinical Research and Project Management Office, The First Hospital of Lanzhou University; f The Fifth Department of General Surgery, The First Hospital of Lanzhou University, Lanzhou, Gansu, China.

**Keywords:** acute cholecystitis, cholecystectomy, endoscopic naso-gallbladder drainage

## Abstract

**Introduction:**

Transitional drainage, which is followed by cholecystectomy plays a key role in the management of acute cholecystitis, especially in high-risk surgical patients. Endoscopic naso-gallbladder drainage (ENGBD) is an alternative to percutaneous transhepatic gallbladder drainage (PTGBD) for patients who need temporary drainage. There is a lack of prospective comparison on the relevant outcomes of the two drainage methods during the period of drainage, especially the subsequent cholecystectomy.

**Methods:**

This is a randomized controlled two-arm non-blind single center trial. Patients with acute cholecystitis undergo emergent or early cholecystectomy and need drainage will be randomly assigned to group PTGBD or ENGBD. Pain score is defined as the primary endpoint, whereas several secondary endpoints, such as the rates of technical success, clinical remission, open conversion of cholecystectomy will be determined to elucidate more detailed differences between two groups. The general feasibility, safety, and quality checks required for high-quality evidence will be adhered to.

**Discussion:**

This study would provide the first type A evidence concerning the comparison of ENGBD versus PTGBD in surgically high-risk patients with acute cholecystitis, it will be the first trial designed to determine the impact of two drainage methods on not only peri-drainage but also peri-LC.

**Trial registration:**

NCT03701464. Registered on October 10, 2018.

## 1 Introduction

Laparoscopic cholecystectomy (LC) was the fundus-first approach for acute cholecystitis (AC). As the global population ages, not only the incidence of AC increases because of the strong relation between AC and age by previous studies,^[1,2]^ but also an increasing number of patients with comorbidities who are poor surgical candidates require precise individualized management. According to Tokyo Guidelines 2018(TG18) flowchart,^[3]^ percutaneous transhepatic gallbladder drainage (PTGBD) should be considered as the first-line alternative to surgical intervention in high-risk patients, especially in some Grade II (moderate) and most of Grade III (severe) AC by the TG18 severity grading.^[4]^ Nevertheless, it may be limited in patients with thrombocytopenia, coagulopathy, large ascitic fluid, and Chilaiditi syndrome.^[5,6]^ In this circumstance, endoscopic gallbladder drainage (EGBD) had also been made another alternative management in high-volume institutes by skilled endoscopists by TG18 management strategies for gallbladder drainage.^[7]^ EGBD, including endoscopic transpapillary gallbladder drainage (ETGBD) by using endoscopic naso-gallbladder drainage (ENGBD) or endoscopic gallbladder stent (EGBS) and endoscopic ultrasound-guided gallbladder drainage (EUS-GBD), is an effective method for acute cholecystitis both technically and clinically and seems to be safer than traditional PTGBD by a systematic review.^[8]^

Despite a prospective study comparing EUS-GBD and PTGBD and a randomized, controlled trial had confirmed that ENGBD and EGBS all appear to be suitable for the drainage^[9]^ so far, there is no prospective comparison of ENGBD or EGBS with PTGBD, especially in their impacts while LC. Temporary or permanent drainage is essential for AC patients at high risk of surgery. In this study, we regard drainage as a temporary bridge to cholecystectomy in patients at high risk of early LC, yet stents were not expected to fall off during the waiting window, we chose this untouched territory comparing the ENGBD and PTGBD.

PTGBD has a technical success rate of nearly 97%, clinical response rates range from 56% to 100%,^[6]^ which is also associated with an overall adverse events rate as high as 14%, including bile leak peritonitis, cholecystitis, bleeding, subcapsular hematoma, pneumothorax, catheter misplacement, and inadvertent removal. Moreover, it is contraindicated in patients with large peritoneal ascites, coagulopathy, thrombocytopenia, and Chilaiditi syndrome.^[5]^ For these reasons, less-invasive forms of ENGBD that are more efficient, more safe and less painful have been developed.^[10]^ It was first proposed in 1984, but reported in 1990,^[11]^ the technical success, clinical success, post-procedure adverse events were 88.9%, 81.5%, 9.3%, respectively, according to the data provided by the only two randomized controlled studies^[9,12]^ in comparing ENGBD and EGBS. Despite such promising achievement from previous studies, the benefits of ENGBD and PTGBD on major clinical outcomes have never been compared prospectively. In particular, the effects of the two drainage method on LC remains uncertain and not only an extensive evaluation but also a retrospective study pointed out that ENGBD was more conducive to the smooth implementation of LC.^[13]^

## 2 Methods

### 2.1 Primary objective

The primary objective of the study is to determine the clinical pain remission of ENGBD versus PTGBD in patients requiring temporary gallbladder drainage, our hypothesis is that in comparison with standard PTGBD, ENGBD could not only relieve the pain symptoms associated with acute cholecystitis itself, but also relatively alleviate the severe pain caused by drainage.

### 2.2 Secondary objectives

The study will also compare ENGBD and PTGBD on clinical outcomes of drainage and the difficulties of cholecystectomy in surgically high-risk patients with acute cholecystitis, especially drainage success rate, clinical remission, and adverse events. Also, their impacts on cholecystectomy will be focused and targeted, including difficulty grading of cholecystectomy, cholecystectomy duration, pathology, and so on.

### 2.3 Trial design

The trial is an investigator-initiated, parallel group, single-center, randomized controlled, with the allocation ration of 1:1, non-blind trial; however, the statistician would not know the grouping.

### 2.4 Participants, interventions, and outcomes

This manuscript written adheres to SPIRIT 2013 Statement.^[14]^

### 2.5 Study setting

**Figure 1:**
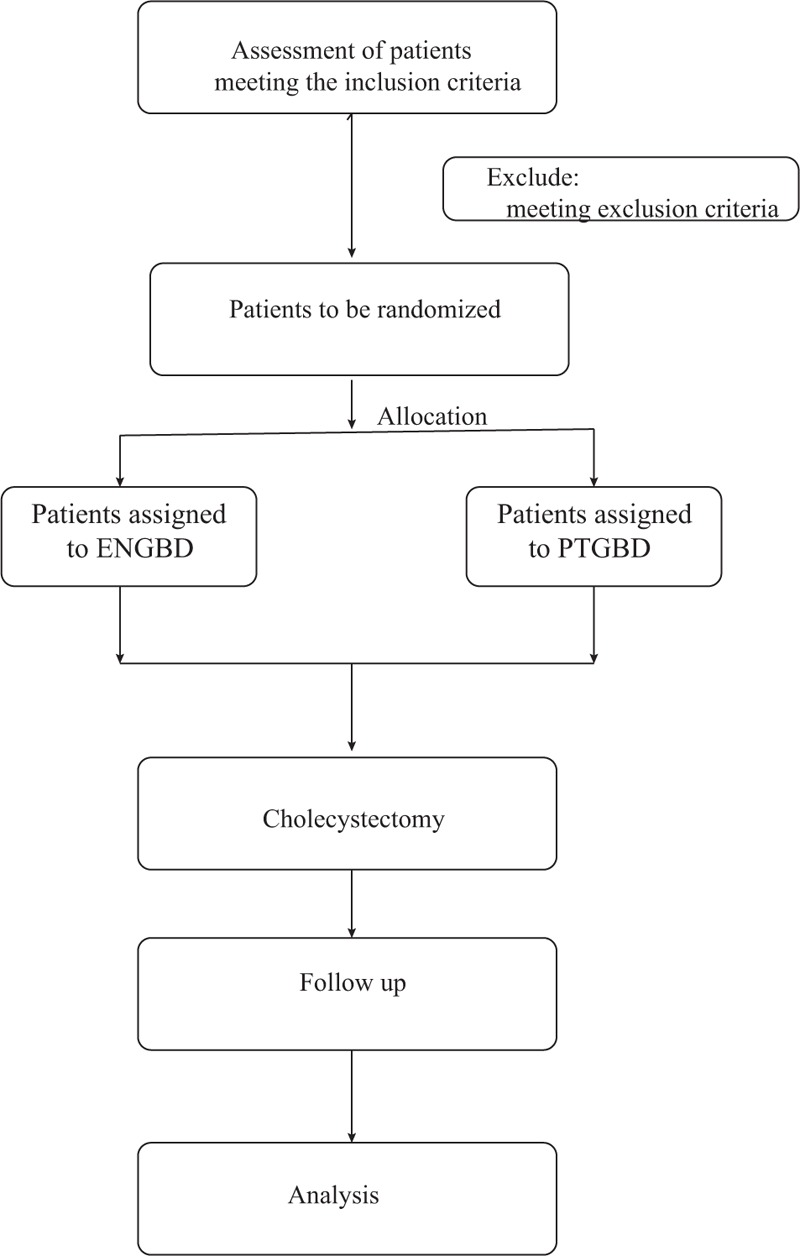
Flow chart of participants.

The trial would be set at an university affiliated tertiary-care A hospital center, which performs approximately more than 2000 Endoscopic Retrograde Cholangiopancreatography (ERCP) procedures per year. The outline of trial procedures is presented in Figure 1.

### 2.6 Eligibility criteria

#### 2.6.1 Inclusion criteria

Patients that were not considered suitable for early or urgent cholecystectomy because of high-surgical risk, in which case biliary drainage followed by delayed LC is recommended by the TG18 flowchart: Grade II (moderate) AC, antibiotics and general supportive care fail to control inflammation; Grade III (severe) AC, after antibiotics and general organ support, negative predictive factors present including jaundice (TBIL ≥2), neurological dysfunction, respiratory dysfunction, or no negative predictive factors present but American Society of Anesthesiologists physical status classification (ASA-PS) is 3 or greater or Charlson comorbidity index (CCI) is 4 or greater.

#### 2.6.2 Exclusion criteria

Exclusion of patients who meet one or more of the following criteria: age <18 or >90 years, pregnant or breastfeeding, severe obesity (body mass index ≥35 kg/m^2^), consent refusal, coagulation dysfunction (INR > 1.5) and low-peripheral blood platelet count (<50 × 10^9^/L), using anticoagulation or antiplatelet drugs; bile duct stones; prior surgery of Bismuth II, Roux-en-Y and choledochojejunostomy, preoperative coexistent diseases: acute pancreatitis, GI tract hemorrhage or perforation, severe liver disease (such as decompensated liver cirrhosis, liver failure, and so on), any malignant diseases.

#### 2.6.3 Endoscopic surgeons criteria

All ERCP operations and LC are completed by an endoscopic surgeons team, with more than 10 years of ERCP and 20 years of LC experience, 400 cases of ERCP and 450 cases of LC per year at present.

### 2.7 Interventions

A systematically trained experienced attending physician assesses that once evidence of gallbladder drainage is available, all patients eligible for inclusion were informed fully of written informed consent, then would be randomly assigned to the interventional group (ENGBD) or to the reference group (PTGBD). Of course, risks and benefits associated with surgery would be routinely communicated and agreed in writing.

The experimental group patients are sedated by intravenous administration of sufentanil and propofol, then selective bile duct cannulation, a 0.025- or 0.035-inch guidewire is advanced into the cystic duct and subsequently into the gallbladder, withdraw the catheter, a 5Fr naso-gallbladder catheter is inserted into the gallbladder along the guidewire. PTGBD is guided by ultrasound, an 18-gauge needle is inserted into the gallbladder, a 0.035-inch guidewire is coiled into the gallbladder and a 9Fr dilator expands the skin, then an 8Fr 20 cm catheter is placed.

When ENGBD was technically unsuccessful or clinically ineffective, endoscopic nasobiliary drainage (ENBD), PTGBD and EUS-GBD were used as alternative procedures. The patients of inclusion would be given appropriate and quality treatment at anytime, in order to reduce waiting and improve adherence. Properly trained volunteers would accompany patients on key treatments, such as ERCP and PTGBD, extubation, LC, etc. This not only improves adherence, because of the convenience to patients, but also monitors adherence to a certain extent.

All patients of inclusion would be assessed by the attending physician in accordance with recent guidelines for AC management, including sufficient infusion, maintenance of electrolyte balance, antibacterial agents, the monitoring of respiratory and hemodynamics, correction of acidosis and complications.

In particular, there is no strict recommendation for extubation time, ENGBD group would extubate when the bile in the naso-gallbladder tube becomes clear, which is approximately 4 days after the drainage based on our experience. PTGBD group extubate after more than 2 weeks due to fistula formation. Also, no consensus has been reached about the optimal timing of LC after drainage. Because most studies^[15–17]^ figure out the short interval increases intraoperative difficulty, we require all patients to undergo cholecystectomy 3 months after drainage, so that edema and inflammation around the gallbladder subsided completely.

### 2.8 Outcome

#### 2.8.1 Primary outcome measures

The primary outcome is the pain score, defined as pain experienced during mobilization from the supine to the standing position. Using the visual-analogue scale, pain score would be obtained within 2 hours before drainage and at post-drainage 24, 48, and 72 hours in conscious and communicating patients by a specially trained nurse, who devoted herself to the objectivity and authenticity of the scores. The details of assessment are as follows: draw a 10-cm line on a piece of paper, mark one end of the line with the number 0, indicating no pain; the other end with 10, indicating the most severe pain; the middle part indicates different degrees of pain. While assessing the pain of patients, make sure the patient could not see the numbers on the paper and let them mark the position according to their feelings about the pain. Then the nurse would get a score based on the mark. Pain scores reflect directly the degree of pain, the remission of pain not only relieves the pain symptoms associated with acute cholecystitis itself, but also relatively alleviates the severe pain caused by tube.

In order to avoid the effects of anesthesia, we chose to evaluate the pain 24 hours after drainage. Especially, all pain assessments should be performed without the administration of pain killers or after using an analgesics for 6 hours. All modifications in pain management are recorded. Our team routinely prepares a multimodal approach to pain management, which is applied mainly based on nonsteroidal anti-inflammatory drugs and other adjuvant drugs, but tries to avoid applying it.

#### 2.8.2 Secondary outcome measures

Table 1 presents the details of secondary outcome measures. These information comprehensively reflect effectiveness and safety of drainage, and difficulties in cholecystectomy.

**Table 1:**
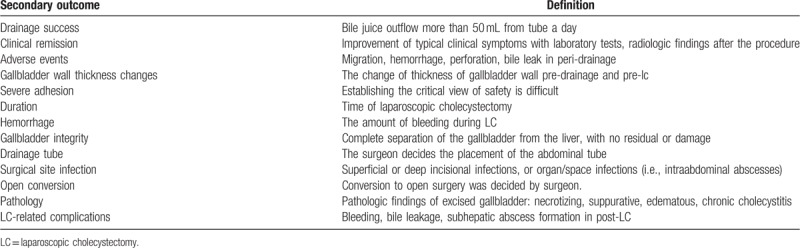
Secondary outcome measures.

### 2.9 Participant timeline

Please see Table 2.

**Table 2:**
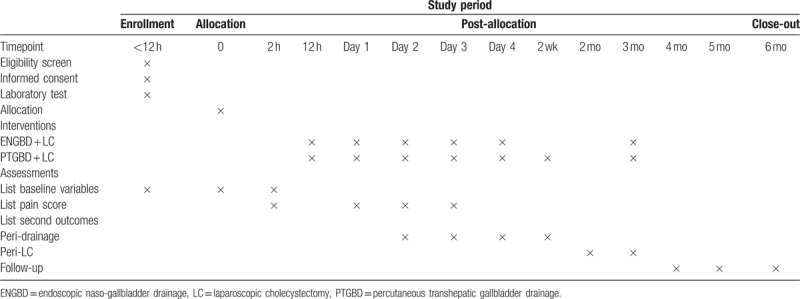
Participant timeline.

### 2.10 Sample size

The sample size calculation is based on the primary outcome-the pain score after drainage. According to the research by Jang et al,^[18]^ using the method provided by Luo et al^[19]^ and Wan et al,^[20]^ the median post-procedure pain score in the PTGBD group was 5.4 ± 3.1. And the mean visual analog score of post-procedure pain in the ENGBD group was 1.3 by Itoi et al.^[9]^ Using a theoretical sample size for two-sample design, nine participants are needed in each group with 80% power and a 5% significance level. Allowing for a 10% loss to follow-up, we plan to recruit 22 patients (11 in each group).

### 2.11 Recruitment

The recruitment would last 20 months, which began in June 2018. This duration was estimated based on the number of admissions for surgical high-risk AC patients at the team in previous 2 years. To achieve adequate participant enrollment, relevant work on the study would be performed around the clock, including nights and weekends as routine clinical practice.

### 2.12 Allocation sequence generation and randomization

The randomization numbers were generated by statistician using computer program with 1:1 allocation, stored and encoded 20 sealed, opaque envelopes independently. When a participant meets inclusion and inclusion criteria, two volunteers first record the name and the code of the next unopened envelope, then open the envelope and sign their names. At last, the treatment assignment would be sent to the attending physicians, which could get only one allocation at a time after all pre-drainage preparations were completed, including the evaluation of patients and informed consent of participants.

### 2.13 Blinding

This is an open-label, unblinded trial for patients and physicians because of the nature of the intervention (position and shape of drainage tube). However, the investigator of collecting secondary outcomes, the assessor of clinical and biological data in charge of statistical analyses and outcome assessment will be masked as to the subjects assigned group.

### 2.14 Data collection, management and analysis

#### 2.14.1 Collection, management

All data are prospectively collected and managed by the well-trained research volunteers.

The baseline data would be collected and registered on inclusion: age, sex, body mass index (BMI), body temperature, interval between onset of AC and drainage, comorbidities and coexisting conditions, american society of anesthesiologists (ASA) grade, charlson comorbidity index (CCI), AC grade based on TG 18 severity grading,^[4]^ white blood cell (WBC) count, electrolytes, total bilirubin, C-reactive protein (CRP), and so on. This would systematically reflect the characteristics of participants.

The peri-drainage data would be obtained from 2 hours before drainage to one week after ENGBD or PTGBD, such as drainage success, clinical remission, adverse events, pain scores within 2 hours before drainage and at post-drainage 24 h, 48 h, 72 h, etc.

The following data are collected and registered on peri-LC: gallbladder wall thickness changes (pre-drainage to pre-LC), duration and hemorrhage of LC, gallbladder integrity, drainage tube, hospitalization (hospital stay after gallbladder drainage and cholecystectomy), surgical site infection, severe adhesion, pathology, and LC-related complications.

Results that are negative or undetected or measured as 0 should have corresponding symbolic representation and can not be vacant, in order to distinguish from missing values. The treatment of outliers should be judged from both medical and statistical aspects by the statistician during blind examination.

Images and biological samples including venous blood and bile will be collected for probably ancillary and long-term research. Also, all patients will be followed up 6 months after cholecystectomy.

The volunteers of data acquisition have rich clinical experience and rigorous working attitude, and received training and guidance. Data storage would be carried out by two independent investigators to ensure reliability and validity.

We would also pay attention to and collect the information of the participants who discontinue or deviate before cholecystectomy and the follow-up are completed. To promote participants’ retention and enthusiasm, we would establish workflow manuals contact patients regularly, communicate properly and take effective care of their health.

#### 2.14.2 Analysis

A predefined statistical analysis plan will be followed. All statistical analyses were conducted with IBM SPSS (version 21.0). *P* values of <.05 were considered to represent statistical significance. Categorical variables would be reported as counts and percentages. The mean and standard deviation or median with interquartile ranges (IQR) were used to describe continuous variable data, as appropriate.

The primary analysis is a comparison between ENGBD and PTGBD for the pain remission based on an intention-to-treat principle. A multilevel, random-slope model would be fitted to the data with time points nested in patients to allow for clustering of data within each patient. This model adjusted for the fixed effects of treatment group, time (−2, 24, 48 , or 72 hours), and treatment × time interaction. An unstructured covariance pattern was selected for the repeated measurements as the least restrictive structure, which resulted in better model fit based on log-likelihood values than more constrained patterns. Estimates of the difference in pain scores between treatment groups were assessed overall and at individual time points.

The secondary analysis is a comparison between ENGBD and PTGBD for the rates of technical and clinical success, adverse events, and changes in the gallbladder wall thickness . Also, the outcomes during cholecystectomy including time duration, open conversion would also be analyzed. The Chi-square test or Fisher exact test was used for dichotomous variables, and Wilcoxon test was used for rank data. For quantitative data, two-tailed Student *t* test is used, if the normal distribution and homogeneous total variance are satisfying, otherwise, Wilcoxon test is used.

A blinded adjudication committee will assess the occurrence of the primary and secondary endpoints after the last patient has completed follow-up.

### 2.15 Data monitoring

Setting up a data monitoring committee and interim safety analysis are unnecessary because of the high security and short cycle of this trial.

### 2.16 Harms

All adverse events thought to be related to the trial would be recorded rigorously and carefully. Any unexpected major serious complications suspected to be associated with ENGBD must be reported to interviewer and attending physicians, the trial may be temporarily stopped, and effective treatment measures in the first place will be taken by attending physicians.

### 2.17 Auditing

A auditing committee consisting of an endoscopist, a surgeon and a sonographer, would monitor the trial at least once every 3 months. They remain independent from investigators and audit the frequency, procedures, and safety of the trial.

### 2.18 Protocol amendments

Any changes to eligibility criteria, outcomes, and analyses would obtain approval from the ethics committee and the clinical trial.

### 2.19 Confidentiality

Data would be handled in a confidential way. During the whole experiment, sensitive information such as the patient name, identity card number would not be exposed by coding.

### 2.20 Access to data

All investigators would have access to the final trial data set after the end of the study.

### 2.21 Dissemination policy

Data analysis, interpretation, and findings would be presented at academic conference and published in peer-reviewed journals. After the end of the trial, the interviewer will complete the writing and submit the manuscript via open access as soon as possible. Every participant would get the results and conclusions by mail.

## 3 Discussion

To our knowledge, the study would be the first randomized controlled study powered to investigate the effect of ENGBD and PTGBD on peri-drainage and peri-LC in poor surgical AC population. Moderate and severe AC are usually associated with increased morbidity and mortality if rushed cholecystectomy is performed, it is becoming increasingly difficult to ignore the optimal management of AC, which includes transitional and permanent drainage, especially when it comes to critically ill patients with multiple comorbidities.

Only one small retrospective discovery of limited quality compared the ENGBD and PTGBD in AC patients to date.^[13]^ Part of the reason may be that ENGBD has to precisely over-select into the difficult visualizing, long, narrow, tortuous cystic ducts, which is a challenge because the ducts could be blocked by stones. The weighted pooled rates for technical success of ENGBD is 81% according to a proportion meta-analysis.^[8]^ However, this technique uses standard off-the-shelf ERCP accessories and can be performed in patients who are coagulopathic or using antiplatelet or anticoagulant agents, and it has certain advantages including better drainage effect which can be observed directly, convenient removal of drainage tube, no establishment of abnormal anatomic fistula, and so on. Thus, it may be regarded as a bridging therapy for acute cholecystitis followed by interval surgical cholecystectomy. Unfortunately, the impact of ENGBD on LC is rarely reported.

Compared to direct surgery, PTGBD shortens the operative duration of LC in patients with moderate to severe AC.^[21]^ However, it has a high risk of conversion to an open procedure during cholecystectomy by a multicenter analysis.^[22]^

As a large surgical endoscopy center that completes more than 2000 ERCPs per year, our team not only has a rich experience in cholecystectomy, but also masters excellently the endoscopic minimally invasive technique, we are confident to complete this major impact study. The trial has the potential to optimize drainage management with direct and realistic benefits for critical AC patients. It is worth mentioning that we would also pay attention to the effects of the two drainage methods on the cholecystectomy. If the study finds out that ENGBD may contribute to late cholecystectomy, it will be an excellent alternative or a powerful complement to PTGBD.

## 4 Trial status

The current study protocol is version 3.0 (May 15, 2019). The trial opened to recruitment on October 10, 2018. Maybe we would complete the recruitment on October 10, 2020.

## Acknowledgments

The authors would like to thank Dr Hao Xu from our medical center for giving us assistance during the therapeutic process.

## Author contributions

All authors approved the final manuscript. PLM and PY make the same contribution to this work. PLM: protocol development, drafting of this manuscript, critical revision of the manuscript for significant intellectual content. PY: Taking responsibility in execution of the trails and patient follow-up. BB, YYL: providing personnel, environmental support and tools and instruments that are vital for the project. HPW, JY: taking responsibility in statistical analysis, logical interpretation and presentation of the results. TYL, YL: reviewing the article before submission not only for spelling and grammar but also for its intellectual content. WBM, XL: constructing an idea or hypothesis for manuscript, planning methodology to reach the conclusion, and providing financial support.

Peilei Mu: 0000-0003-3513-9513

Ping Yue: 0000-0001-8736-949X

Bing Bai: 0000-0001-8672-8569

Yanyan Lin: 0000-0001-7417-0190

Jinduo Zhang: 0000-0002-3425-9614

Haiping Wang: 0000-0002-7771-5077

Ying Liu: 0000-0002-0997-4480

Jia Yao: 0000-0002-0811-8287

Wenbo Meng: 0000-0002-9355-0225

Xun Li: 0000-0003-3787-1558

## References

[1] Parmar AD Sheffield KM Adhikari D PREOP-gallstones: a prognostic nomogram for the management of symptomatic cholelithiasis in older patients. Ann Surg 2015;261:1184–90. 25072449 10.1097/SLA.0000000000000868 PMC4309752

[2] Cho JY Han HS Yoon YS Risk factors for acute cholecystitis and a complicated clinical course in patients with symptomatic cholelithiasis. Arch Surg 2010;145:329–33. [discussion 333]. 20404281 10.1001/archsurg.2010.35

[3] Okamoto K Suzuki K Takada T Tokyo Guidelines 2018: flowchart for the management of acute cholecystitis. J Hepatobiliary Pancreat Sci 2018;25:55–72. 29045062 10.1002/jhbp.516

[4] Yokoe M Hata J Takada T Tokyo Guidelines 2018: diagnostic criteria and severity grading of acute cholecystitis (with videos). J Hepatobiliary Pancreat Sci 2018;25:41–54. 29032636 10.1002/jhbp.515

[5] Itoi T Sofuni A Itokawa F Endoscopic transpapillary gallbladder drainage in patients with acute cholecystitis in whom percutaneous transhepatic approach is contraindicated or anatomically impossible (with video). Gastrointest Endosc 2008;68:455–60. 18561927 10.1016/j.gie.2008.02.052

[6] Winbladh A Gullstrand P Svanvik J Systematic review of cholecystostomy as a treatment option in acute cholecystitis. HPB (Oxford) 2009;11:183–93. 19590646 10.1111/j.1477-2574.2009.00052.x PMC2697889

[7] Mori Y Itoi T Baron TH Tokyo Guidelines 2018: management strategies for gallbladder drainage in patients with acute cholecystitis (with videos). J Hepatobiliary Pancreat Sci 2018;25:87–95. 28888080 10.1002/jhbp.504

[8] Khan MA Atiq O Kubiliun N Efficacy and safety of endoscopic gallbladder drainage in acute cholecystitis: is it better than percutaneous gallbladder drainage? Gastrointest Endosc 2017;85: 76–83.e3. 10.1016/j.gie.2016.06.032 27343412

[9] Itoi T Kawakami H Katanuma A Endoscopic nasogallbladder tube or stent placement in acute cholecystitis: a preliminary prospective randomized trial in Japan (with videos). Gastrointest Endosc 2015;81:111–8. 25527052 10.1016/j.gie.2014.09.046

[10] Kedia P Sharaiha RZ Kumta NA Endoscopic gallbladder drainage compared with percutaneous drainage. Gastrointest Endosc 2015;82:1031–6. 25952093 10.1016/j.gie.2015.03.1912

[11] Feretis CB Manouras AJ Apostolidis NS Endoscopic transpapillary drainage of gallbladder empyema. Gastrointest Endosc 1990;36:523–5. 2227333 10.1016/s0016-5107(90)71134-0

[12] Yang MJ Yoo BM Kim JH Endoscopic naso-gallbladder drainage versus gallbladder stenting before cholecystectomy in patients with acute cholecystitis and a high suspicion of choledocholithiasis: a prospective randomised preliminary study. Scand J Gastroentero 2016;51:472–8. 10.3109/00365521.2015.1115116 26595503

[13] Toyota N Takada T Amano H Endoscopic naso-gallbladder drainage in the treatment of acute cholecystitis: alleviates inflammation and fixes operator's aim during early laparoscopic cholecystectomy. J Hepatobiliary Pancreat Surg 2006;13:80–5. 16547666 10.1007/s00534-005-1062-4

[14] Chan AW Tetzlaff JM Altman DG SPIRIT 2013 statement: defining standard protocol items for clinical trials. Ann Intern Med 2013;158:200–7. 23295957 10.7326/0003-4819-158-3-201302050-00583 PMC5114123

[15] Inoue K Ueno T Nishina O Optimal timing of cholecystectomy after percutaneous gallbladder drainage for severe cholecystitis. BMC Gastroenterol 2017;17:71. 28569137 10.1186/s12876-017-0631-8 PMC5452332

[16] Han IW Jang JY Kang MJ Early versus delayed laparoscopic cholecystectomy after percutaneous transhepatic gallbladder drainage. J Hepatobiliary Pancreat Sci 2012;19:187–93. 21938408 10.1007/s00534-011-0458-6

[17] Choi JW Park SH Choi SY Comparison of clinical result between early laparoscopic cholecystectomy and delayed laparoscopic cholecystectomy after percutaneous transhepatic gallbladder drainage for patients with complicated acute cholecystiti. Korean J Hepatobiliary Pancreat Surg 2012;16:147–53. 26388926 10.14701/kjhbps.2012.16.4.147 PMC4575000

[18] Jang JW Lee SS Song TJ Endoscopic ultrasound-guided transmural and percutaneous transhepatic gallbladder drainage are comparable for acute cholecystitis. Gastroenterology 2012;142:805–11. 22245666 10.1053/j.gastro.2011.12.051

[19] Luo D Wan X Liu J Optimally estimating the sample mean from the sample size, median, mid-range, and/or mid-quartile range. Methods Med Res 2018;27:1785–805. 10.1177/0962280216669183 27683581

[20] Wan X Wang W Liu J Estimating the sample mean and standard deviation from the sample size, median, range and/or interquartile range. BMC Med Res Methodol 2014;14:135. 25524443 10.1186/1471-2288-14-135 PMC4383202

[21] Lee R Ha H Han YS Percutaneous transhepatic gallbladder drainage followed by elective laparoscopic cholecystectomy for patients with moderate to severe acute cholecystitis. Medicine (United States) 2017;96:10.1097/MD.0000000000008533 PMC5682837 29095318

[22] Sanjay P Mittapalli D Marioud A Clinical outcomes of a percutaneous cholecystostomy for acute cholecystitis: a multicentre analysis. HPB (Oxford) 2013;15:511–6. 23750493 10.1111/j.1477-2574.2012.00610.x PMC3692020

